# Evaluating Delivery of a CBT-Based Group Intervention for Schoolchildren With Emotional Problems: Examining the Reliability and Applicability of a Video-Based Adherence and Competence Measure

**DOI:** 10.3389/fpsyg.2021.702565

**Published:** 2021-06-28

**Authors:** Lene-Mari Potulski Rasmussen, Joshua Patras, Bjørn Helge Handegård, Simon-Peter Neumer, Kristin Dagmar Martinsen, Frode Adolfsen, Anne Mari Sund, Monica Martinussen

**Affiliations:** ^1^Regional Centre for Child and Youth Mental Health and Child Welfare, Faculty of Health Sciences, UiT the Arctic University of Norway, Tromsø, Norway; ^2^Centre for Child and Adolescent Mental Health, Oslo, Norway; ^3^Department of Psychology, Faculty of Social Sciences, University of Oslo, Oslo, Norway; ^4^Regional Centre for Child and Youth Mental Health and Child Welfare, Department of Mental Health, Faculty of Medicine and Health Sciences, NTNU - Norwegian University of Science and Technology, Trondheim, Norway; ^5^Department of Child and Adolescent Psychiatry, St. Olavs University Hospital, Trondheim, Norway

**Keywords:** youths, emotional problems, program fidelity, reliability, applicability, adherence, competence, group intervention

## Abstract

Adherence and competence are essential parts of program fidelity and having adequate measures to assess these constructs is important. The Competence and Adherence Scale for Cognitive Behavioral Therapy (CAS CBT) was developed to evaluate the delivery of cognitive therapies for children with clinical anxiety. The present study is an assessment of the slightly adapted version of the CAS CBT evaluating the delivery of a Cognitive Behavioral Therapy (CBT)-based preventive group intervention: EMOTION: *Kids Coping with Anxiety and Depression*. This study was part of a Norwegian cluster randomized controlled trial (cRCT) investigating the effectiveness of a transdiagnostic intervention, the EMOTION program—an indicated prevention program targeting anxious and depressive symptoms. The applicability and psychometric properties of the CAS CBT were explored. Results are based on six raters evaluating 239 video-recorded sessions of the EMOTION program being delivered by 68 trained group leaders from different municipal services. Interrater reliability (intraclass correlation coefficients, ICC [3, 1]) indicated fair to good agreement between raters. Internal consistency of the instrument's key domains was calculated using the Omega coefficient which ranged between 0.70 to 0.94. There was a strong association between the two scales Adherence and Competence, and inter-item correlations were high across the items, except for the items rating the adherence to the session goals. Competence and Adherence Scale for Cognitive Behavioral Therapy is a brief measure for use in first-line services, with some promising features for easily assessing program fidelity, but some of the results indicated that the instrument should be improved. Future attention should also be made to adapt the instrument to fit better within a group setting, especially regarding evaluation of session goals. More research on how to adequately evaluate fidelity measures are also warranted.

**Clinical Trial Registration:** www.ClinicalTrials.gov, identifier: NCT02340637.

## Introduction

Manual-based interventions consist of prescribed procedures with specified goals and activities designed to produce changes in the target group. Treatment fidelity (also known as treatment integrity or program fidelity) may be viewed as a multidimensional construct, which broadly reflects whether an intervention is delivered as originally planned (Perepletchikova and Kazdin, 2005; McLeod et al., 2009; Gresham, 2014). Following the program's core components is considered necessary to produce the desired outcomes (Bond et al., 2001; Dusenbury et al., 2003). This is generally referred to as adherence and reflects the therapists' utilization of the prescribed intervention procedures (Southam-Gerow et al., 2016). Another important part of program delivery is competence, which represents the therapists' quality of delivery, and how well the intervention is conducted (Perepletchikova and Kazdin, 2005; McLeod et al., 2018). Other aspects of treatment integrity, such as differentiation (if and how treatment differs from others), dosage (length and frequency), and participant responsiveness (benefits for the participants) have also been considered as important factors of program delivery (Waltz et al., 1993; Dane and Schneider, 1998; Perepletchikova and Kazdin, 2005).

Although treatment integrity is considered a multidimensional construct, adherence, and competence comprise the most common and most important dimensions of treatment fidelity and have so far generated the greatest amount of interest regarding assessment and monitoring of manualized therapies (Perepletchikova and Kazdin, 2005; Hogue et al., 2008). According to the literature, adherence does not necessarily require competence, but competence will always be presupposed by adherence (McGlinchey and Dobson, 2003; Perepletchikova et al., 2007). This implies that delivery of an intervention may be adherent, but incompetently performed. Despite the conceptual difference between adherence and competence, the constructs overlap considerably, and both constructs are considered central during program delivery. As such, a high degree of adherence and competence to an effective program is associated with better treatment outcomes (Perepletchikova and Kazdin, 2005; Carroll et al., 2007).

Measures targeting these constructs are still scarce, particularly in the field of child psychotherapy (McLeod et al., 2009; Southam-Gerow and McLeod, 2013), and fidelity in general has received less attention in treatment studies compared to the effectiveness of the intervention (Perepletchikova et al., 2007). One reason could be that the operational definition and components of specific interventions are different, as well as the requirements for implementation (Perepletchikova et al., 2007). Having fidelity measures that embrace specific parts of the intervention make it difficult to compare with other measures, while more generic instruments might not capture the essential elements of an evidence-based intervention (Calsyn, 2000; Perepletchikova et al., 2007). As such, developing instruments which targets both the unique dimensions (e.g., core ingredients of an intervention) and non-specific dimensions [e.g., frame/structure of Cognitive Behavioral Therapy (CBT)-principals] of the interventions are beneficial. It is therefore desirable to develop adequate measures that asses both adherence and competence, in addition to treatment outcome when evaluating a manual-based intervention. These elements are also important in implementation research because they indicate how well staff have been trained and supported to use a given intervention (Carroll et al., 2007).

Commonly used methods to assess program fidelity are self-reports and observations of the sessions. In the field of CBT, some self-report measures have been developed which have the advantages of being easier to administer and less resource demanding than observations, such as, the Cognitive Behavioral Therapy Checklist (CBTC; Kendall et al., 2001). Filling out self-reports and checklists following delivery can also serve as a reminder to interventionists about program contents, which in turn can serve to reinforce the use of intervention core components (Bellg et al., 2004). Self-reports, however, rely on individuals' ratings of their own performance, which allows for potential reporter bias (Bellg et al., 2004). Observations, by contrast, are conducted by third parties and are therefore considered a more rigorous and objective measure of treatment adherence and competence (Hogue et al., 1996), though more costly and time consuming.

According to the literature, few such measures for CBT-based interventions with children exist (Southam-Gerow and McLeod, 2013; McLeod et al., 2018), particularly observation tools. There are even fewer studies examining and reporting the psychometric properties on measures evaluating adherence and/or competence during delivery of CBT for children and adolescents (Rapley and Loades, 2019). For the instruments that do exist, there are variations on how these are designed, both in terms of structure and content. Some only assess adherence (Gutermann et al., 2015; Southam-Gerow et al., 2016), whereas others assess only competence (Stallard et al., 2014; Gutermann et al., 2015; McLeod et al., 2018). There are a few measures assessing both adherence and competence (Hogue et al., 2008; Bjaastad et al., 2016; Smith, 2017). All these measures address CBT for anxious youth in some way (both clinical and non-disorder), except for one, which is aimed at CBT for substance abuse (Hogue et al., 2008). None of these studies, investigates adherence or competence in a prevention setting, nor within a group format. Hence, to guide the field forward, it is important to continue to develop measures addressing the key dimensions of fidelity, and investigate the psychometric properties and applicability of these measures (McLeod et al., 2009).

To ensure that the instrument used can be applied to similar, but still different contexts, investigating the instruments is important. For instance, CBT-based interventions for indicated prevention share many common features with clinical therapy; however, conducting interventions in the prevention field involves several unverifiable factors (e.g., undefined symptoms in the children, scheduling issues, etc.). Also, resources aligned to support implementation are often limited (Forman et al., 2009), and typically, assessing adherence and competence is often omitted from prevention studies (Cross and West, 2011; Bumbarger, 2014). Observations of fidelity are particularly rare given the extra resources needed (Hogue et al., 1996; Schoenwald et al., 2011). Further, although highly educated and experienced within their field, many of the employees working in prevention services and delivering interventions do not have prior training in CBT. Also, group interventions have many advantages (e.g., sharing problems, reaching more children at the same time), but different group sizes, group dynamics, or other issues during delivery may occur. All of these matters may impact delivery of a CBT-based program, and further justify the need to measure fidelity for these interventions.

According to researchers in the field (Perepletchikova et al., 2007; Southam-Gerow and McLeod, 2013), treatment integrity needs further elaboration, particularly regarding development and validation of measures. Normally, investigating whether a test measures what is intended (construct validity), confirmatory factor analysis (CFA; Floyd and Widaman, 1995) is often applied. For treatment integrity measures, however, this could introduce some challenges, especially for observational measures. This is because fidelity, and thus the instrument structure may be influenced by the study setting and the individuals involved (e.g., therapists and/or clients) (Perepletchikova and Kazdin, 2005; Allen et al., 2018) meaning that a factor analysis could provide an overall factor based on given items, however they may not be psychometrically meaningful (Gresham, 2014).

In relation to this, the term “flexibility within fidelity” (Kendall et al., 2008) has been gaining increased attention in manual-based interventions, referring to the group leaders' ability to deliver the intervention adherently (providing the core ingredients), while at the same time being flexible when adapting them to the context (i.e., considering individual differences among the children).

Developing measures that can capture the different aspects of the intervention being delivered serves interest. Including both the non-specific dimensions related to the program structure, such as CBT principals in general, as well as the more intervention specific domains of the program (e.g., specific goals for the sessions) increases the possibility of using the same measure to compare treatment fidelity across settings and similar, but different, treatment procedures (Calsyn, 2000). Such measures will be easier to implement and administer, and less time-consuming compared to rating each session separately (Gutermann et al., 2015).

Competence and Adherence Scale for Cognitive Behavioral Therapy (CAS CBT; Bjaastad et al., 2016) is a new observation-based measure, which is designed for assessing the degree of adherence and competence during therapy on youths with anxiety disorders. The instrument was inspired by inspired the Cognitive Therapy Adherence and Competence Scale (CTACS; Barber et al., 2003), which is a similar instrument used in adult CBT therapy. Thus, the development of this measure is based upon previous work regarding delivery of CBT therapy assessments. Further, in line with the program developers (Bjaastad et al., 2016), this current study has also used Perepletchikova and Kazdin (2005); Perepletchikova et al. (2007) work on treatment integrity, to conceptualize and frame adherence and competence. The instrument design makes it applicable to other CBT-interventions, particularly targeting emotional problems. Anxiety and depression in children are among the most prevalent psychological problems (Merikangas et al., 2009), and structured CBT interventions are commonly used to address these mental health problems (Crowe and McKay, 2017). The CAS CBT has previously been used with trained therapists, working in outpatient clinics treating youth with clinical anxiety (Wergeland et al., 2014; Bjaastad et al., 2016). However, research indicate that many children with emotional problems are being overlooked, and not receiving the mental health care they need (Stallard et al., 2008; Sund et al., 2011). Prevention is therefore essential to target these issues, before they develop into mental disorders (Georgiades et al., 2006; Kovacs and Lopez-Duran, 2010) and early interventions are becoming an important part of municipal services for children. Research shows, however, that prevention programs are implemented with a lack of fidelity given that delivery are rarely monitored (Bumbarger, 2014). When moving efforts from specialist care to first line services, it is evident to assess that the interventions are conducted as described by program developers. Having a brief measure to assess if manual-based CBT-interventions are delivered as intended, and how they are conducted will provide insightful knowledge regarding use of such programs. Thus, the main goal of the current study was to investigate the reliability of the CAS CBT (Bjaastad et al., 2016) and to consider the applicability of the measure within a prevention setting.

Previous research on CAS CBT has primarily been conducted by the instrument developers (Bjaastad et al., 2016), who performed an exploratory factor analysis (EFA) identifying two factors: (1) CBT structure and session goals and (2) Process and relational skills in a sample of *N* = 182 youths (*M* age = 11.5 years, *SD* = 2.1). The first factor loaded on the items assessing how the sessions was conducted in relation to general CBT principals (items 1–4), and the goals for the session (items 9–10). The second factor included the items 4–7, which assesses positive reinforcement, collaboration, and flexibility. These two scales also showed good internal consistency (α = 0.87 and α = 0.89, respectively). The CAS CBT also showed good to excellent interrater reliability [intraclass correlation coefficients (ICC) = 0.83 for Adherence and 0.64 for Competence; Cicchetti, 1994] and high rater stability with an ICC = 0.89 for Adherence and 0.92 for Competence when the videos were rescored after an average of 17.4 months (Bjaastad et al., 2016). Besides this study, three other studies (Villabø et al., 2018; Harstad et al., 2021; Jeppesen et al., 2021) have used CAS CBT to evaluate therapist adherence and competence within a clinical setting. The sample in Jeppesen et al. (2021) was *N* = 396 youths (*M* age = 10.3, *SD* = 2.4), and in Villabø et al. (2018) *N* = 165 children; ages 7–13 years were included. However, limited information regarding the instrument psychometrics was presented. In the study by Harstad et al. (2021) including *N* = 165 (*M* age, 10.46, *SD* = 1.49), the psychometric properties of CAS CBT in a naturalistic treatment setting was explored. They found an excellent internal consistency (Cronbach's alpha = 0.88), and the EFA identified the same two factors as Bjaastad et al. (2016). To our knowledge, there are no other studies assessing group leader's adherence and competence using CAS CBT when running an indicated prevention program both for anxious and sad children, in municipal services (e.g., non-clinical settings). Our research questions were therefore: What are the psychometric properties (e.g., reliability) and how does the instrument apply in a preventive group-based setting, targeting both anxiousness and sadness in young children. Considering the format of the instrument, which can easily be transferred and applied to other interventions, the developers of the CAS CBT also highlighted a need to independently validate the instrument using manualized interventions targeting related problem areas, but with different delivery modalities and target groups (Bjaastad et al., 2016).

## Methods

This study was part of a Norwegian multi-site cluster randomized controlled trial (cRCT), investigating the effectiveness and the implementation of the EMOTION program (Patras et al., 2016). The RCT trial recruited 36 schools from three regions in Norway (South-East, Mid, and North), which were randomized to intervention (including *N* = 266 children) or control (including *N* = 428 children). EMOTION: *Kids Coping with Anxiety and Depression* (Martinsen et al., 2014), is a group-based preventive CBT intervention for children with elevated levels of anxious and/or depressive symptoms. The intervention is run in a school setting by group leaders from different municipal services (e.g., school mental health service). The maximum number of children in each intervention group was seven, therefore 71 children were randomly excluded from the study due to a lack of group leaders to conduct groups, explaining some of the discrepancy between intervention and control group. Ethical approval was obtained from The Regional Committee for Health and Medical Research Ethics (2013/1909/REK Sør-Øst), and the study was registered in clinical trials (NCT02340637).

### Participants

Participants were trained group leaders (*N* = 68) with a mean age of 39.6 (*SD* = 9.7 years, 94% women) delivering the EMOTION program. The study sample were psychologists/specialists (35%), school health nurses (14%), educational and psychological counselors (18%), educators (11%), child-care workers (6%), occupational therapists (3%) as well as psychology students (5%), and 8% “others” (e.g., counselor, project leader etc.). Almost 70% of the participants had former experience working with anxiety and depression in youths, and 38% had previously used CBT. They received a 3-day training, with 1-day introduction in general CBT, followed by a 2-day workshop in the specific program components of the EMOTION program. Each day of training lasted approximately 6 h. During delivery of the intervention, the group leaders received weekly supervision from trained CBT supervisors. The supervisors also received supervision from the program developers.

The municipals and interested schools were informed about the study by the local research staff in each region and signed an agreement with the project if they wanted to participate. The 36 participating schools across the country (both rural and urban) were then paired with another school in the same region, before they were randomly assigned to one intervention and one control school throughout the study. The children were recruited from the participating schools, by receiving information about the study. All children who had a signed consent from parents, underwent screening at school using the Multidimensional Anxiety Scale for Children (MASC-C; March et al., 1997) and The Mood and Feelings Questionnaire-short version (SMFQ; Angold et al., 1995). Based on scores above a predetermined cutoff on anxiety and/or depression, the children received an invitation to participate in the study if they scored one *SD* above the cut-off (based on a population mean) on anxiety, depression, or both. Parents were included if the children agreed to participate. The children (*N* = 266) in the active arm of RCT study undergoing the EMOTION program had a mean age of 9.64 years (*SD* = 0.93), where 56.9% were girls. More than 95% of the children were Norwegian, Nordic or of Western European origin.

### The EMOTION Intervention

The EMOTION program (Martinsen et al., 2014) is aimed at reducing symptoms of anxiety and depression in children 8–12 years. The transdiagnostic intervention builds on CBT principles, and during the 20 sessions (1-h sessions, twice per week), the main goals were to teach children different sets of skills and strategies to handle their anxious or sad feelings. Thus, each session was built upon a regular CBT structure (e.g., checking homework, putting up an agenda) and intervention specific topics (e.g., problem solving, behavioral experiments). Additionally, parents received a seven-session course where the children also attended four of these sessions. The parent sessions focused on positive parenting. Parents were also introduced to the same skills as the children learned in their groups and were also taught how to support the child when approaching feared and avoided activities and help to raise their moods. Two group leaders trained in the intervention led each group, both child and parent sessions. They did not have differentiated roles (e.g., no primary or secondary leader), therefore creating a dyad of individuals. Previous studies have found a significant reduction in anxious and depressed symptoms (Martinsen et al., 2019), and at 12-month follow-up, results were still significant for anxiousness (Loevaas et al., 2020). The EMOTION intervention also seems to have a positive effect on emotional regulation skills (Loevaas et al., 2018), and children's quality of life and self-esteem (Martinsen et al., 2021), as reported by the children.

### Procedure

The research staff distributed video cameras to the intervention group leaders before starting new groups with a list of which sessions to record. A block of four consecutive child sessions and two consecutive parent sessions were chosen for each group. The first session of each session-block was chosen randomly to get coverage of a variety of sessions. Sessions were chosen in blocks to simplify the data collection for the group leaders. For example, a group leader may have been randomly assigned to start with session 10, and then follow with sessions 11, 12, and 13. The first and the last session of the program were excluded from the fidelity checks due to the content (introduction and finalization of the groups, respectively). When the groups were finished, the project staff collected and stored the video files at a secure server at one of the participating sites.

### Measure

The CAS CBT consists of 11-items, built upon three main sections, which cover the key domains in CBT for children with anxiety (Bjaastad et al., 2016). The instrument is free to use and can be downloaded with the scoring instructions at https://www.kognitiv.no/utdanning-i-kognitiv-terapi/terapeutiske-hjelpemidler/barn-unge/.

The instrument allows scoring of “*Cognitive behavior therapy structure*” (e.g., homework, session structure, and progress), “*Process- and relational skills*” (e.g., reinforcement, collaboration, and flexibility), and “*Facilitating and completing session goals*” (specific goals for the session based on the treatment protocol). Adherence is assessed by different items within each of the main sections (e.g., homework, session structure, and progress), while competence is scored globally for each of the main sections. This means that the competence item “Cognitive therapy structure” includes an overall competence assessment of both homework and session structure/progress. Further, the item “Flexibility” is rated as a competence score. In addition, there are two questions assessing the overall adherence and competence of the session. These are scored globally and were added as supplementary items to the scale. The adherence score was rated from 0 = None to 6 = Thorough, where all the even numbers had a descriptor. The competence score ranges from 0 (Poor skills) to 6 (Excellent skills), with an explanation attached to the ratings, for the indicators 0, 2, 4, and 6, describing different qualities which needed to be fulfilled. The odd numbers (1, 3, and 5) do not provide a unique behavioral indicator and are interpreted as a score between the different scores following an explanation. Furthermore, there are two questions about the video quality and challenges with the scoring (e.g., “Where there any scoring difficulties due to quality of the videotape?”).

In this study, we made a few adaptations of the instrument to fit the EMOTION program in collaboration with the CAS CBT developer. In the original CAS CBT, the parents were included with one item called “parental involvement” (Bjaastad et al., 2016). In EMOTION, the parents received seven sessions and therefore this item was removed. The seven parent sessions were rated separately with the same structure as the CAS CBT for children. Also, in the original version, there were two program goals to be rated, but in our version, we had up to three goals, so one item was added. The instrument developer(s) approved the modifications.

#### The Scoring Team

The scoring team consisted of six people, including a researcher with previous experience using the instrument, and students with a master's degree or higher in psychology or childcare. The scoring team received 1 day of training (6 h) by the instrument developer in the core elements of the scoring instrument (CAS CBT). In addition, they received a 2-day training, which lasted about 4 h each, in the EMOTION program; similar to the group leader training, focusing on key aspects of the program, session by session. Prior to start up, the raters had to score the same three videos for training purposes and checking for interrater reliability (ICC). If consensus was met with the expert rater, they could continue. The experienced researcher, with previous clinical practice and video rating experience, was the expert rater whom the other raters were tested against. The expert rater scored 40 videos individually and 66 videos for interrater reliability (ICC). Additionally, the team had regular meetings to calibrate, reach consensus and avoid drift. During these meetings, the team scored the same video beforehand, and then met to discuss the results and solve any disagreements. The raters received randomly assigned video recordings for scoring provided by a research coordinator. All raters signed a declaration of confidentiality. Altogether, a total of *N* = 239 sessions (17% of all sessions) were recorded and scored for *N* = 52 groups (170 child sessions and 69 parent sessions). During the project period, ongoing reliability tests were conducted which resulted in 66 randomly selected videos (28%) used for testing interrater reliability (See Table 1 for an overview). Furthermore, raters were trained and instructed by the instrument developer to score the group leaders as a unit, creating an overall score of the two group leaders' adherence and competence delivered during the session. Thus, if one of the group leaders demonstrated a lower level of competence, this would reduce the overall competence score due to its impact on the overall performance.

**Table 1:** Distribution of videos per observer (single scored and ICC).

	**Observer**						
	**Expert**	**R1**	**R2**	**R3**	**R4**	**R5**	**Total**
Single videos scored	40	37	22	82	27	31	239
Videos used for ICC (%)		19 (51%)	10 (45%)	15 (18%)	12 (44%)	10 (32%)	66 (28%)
ICC adherence		0.67	0.54	0.83	0.69	0.86	
ICC competence		0.45	0.45	0.63	0.58	0.53	

*ICC, intraclass correlation [3, 1] by Shrout and Fleiss (1979); two-way mixed effect model, single measurement (absolute agreement); R1, rater 1; R2, rater 2; R3, rater 3; R4, rater 4; R5, rater 5*.

### Statistical Analyses

#### Interrater Reliability

The reliability analyses and descriptive analyses were conducted using SPSS statistical packages (24.0). Interrater reliability between raters was calculated using intraclass correlations (ICC, [3, 1]; Shrout and Fleiss, 1979). The ICCs were calculated by using the model [3, 1] with absolute agreement, which is a Two-Way Mixed Effects Model where people effects are random and measures effects are fixed. The videos were scored by the expert rater and compared against the other observers using the single measure option. The ICC is interpreted as the proportion of the total variance that is between sessions. Results were interpreted using Cicchetti (1994) principles where ICCs <0.40 is considered poor agreement, ICCs between 0.40 to 0.59 indicate fair agreement, ICCs between 0.60 to 0.74 reflect good agreement and ICCs >0.75 show excellent agreement.

#### Internal Consistency

Given that the items are ordinal, reliability in terms of internal consistency for the total scale as well as the different subscales (key domains) was calculated using the Omega coefficient, including the 95% confidence interval (McDonald's Omega; McDonald, 1999). Omega if item deleted was also included. Similarly as with Cronbach's alpha, an Omega coefficient above 0.70 is considered acceptable (EFPA, 2013; Watkins, 2017).

#### Correlations

Inter-item correlations between the items were computed using polychoric correlations (Jin and Yang-Wallentin, 2017), which consider the ordinal measurement level of the Likert-scale and interpreted similarly as Person's *r*. Correlations between the global adherence and mean of the seven adherence items, and between the global competence score and mean of the remaining four competence items, as well the adherence and competence total scores were computed using Pearson's *r*.

## Results

Approximately 20% (*N* = 267) of the total number of sessions were video recorded and intended to be scored using the slightly modified version of CAS CBT (Bjaastad et al., 2016). However, some of the videos could not be scored (e.g., only parts of the session were recorded due to technical issues, poor video quality or camera placement made scoring impossible). This resulted in 239 (17 %) individually recorded child and parent sessions for 52 groups (*M* = 3.0, *SD* = 1.61 sessions per group). The items generally displayed a symmetric distribution of the response categories, except for items assessing the adherence of the session goals (item 8, 9, and 10). Those showed a positively skewed distribution (on a scale from 0 = None to 6 = Thorough), with 35–60% of the responses falling in response category 0 (not present).

### Interrater Reliability

Results showed fair to good interrater reliability (from ICC = 0.40 to 0.74) on all items, and on the mean adherence and mean competence score across all raters compared with the expert rater. See Table 2 for a complete overview of the Mean (*SD*), and ICC scores between the expert rater and the student raters. In general, the ICC scores were in the lower range, where the items reflecting process and relational skills received the lowest scores (0.42–0.52). This indicates that there were some issues assessing group leaders' adherence and competence, and that the items reflecting relational skills were more difficult for the raters to evaluate and agree upon.

**Table 2:** Inter-rater reliability between expert and student raters for the 11-item CAS CBT scale and mean adherence/competence.

**Item/Variable**	***M*** **(*****SD*****)**			**ICC**
	**Total**	**Expert rater**	**Student raters (*n* = 5)**	
***N*** **videos**	***n*** **=** **239**^**a**^	***n*** **=** **66**^**b**^		
**CBT structure**				
1. Homework review/planning homework (adherence)	3.46 (1.97)	4.00 (2.05)	3.21 (2.07)	0.60
2. Progress and structure (adherence)	3.59 (1.52)	3.20 (1.69)	3.12 (1.66)	0.60
3. Cognitive therapy structure (competence for items 1–2)	3.36 (1.48)	3.29 (1.74)	3.03 (1.49)	0.52
**Process/Relational skills**				
4. Positive reinforcement (adherence)	3.91 (1.32)	3.83 (1.47)	3.55 (1.54)	0.48
5. Collaboration (adherence)	4.06 (1.38)	4.24 (1.18)	3.83 (1.38)	0.40
6. Flexibility (competence)	4.00 (1.36)	4.15 (1.26)	3.64 (1.44)	0.42
7. Process and relational skills (competence for items 4–6)	3.90 (1.32)	4.23 (1.25)	3.44 (1.42)	0.52
**Facilitating and completing session goals**				
8. Session goal 1 (adherence)	3.53 (1.61)	3.15 (2.12)	3.15 (1.85)	0.63
9. Session goal 2 (adherence)	2.93 (2.10)	2.58 (2.32)	2.82 (2.15)	0.74
10. Session goal 3^c^ (adherence)	2.61 (1.95)	1.65 (1.60)	1.68 (1.67)	0.55
11. Session goals (competence for items 8–10)	3.19 (1.47)	3.08 (1.76)	2.75 (1.49)	0.56
**Overall evaluation**				
12. Global adherence	3.60 (1.47)	3.18 (1.87)	3.23 (1.37)	0.49
13. Global competence	3.60 (1.40)	3.55 (1.38)	3.21 (1.37)	0.51
Mean score adherence (7 items)	3.55 (1.24)	3.43 (1.33)	3.19 (1.23)	0.60
Mean score competence (4 items)	3.61 (1.26)	3.69 (1.34)	3.22 (1.30)	0.60

*Total scale (11 items); The adherence score was rated from 0 = None to 6 = Thorough. The competence score ranges from 0 (Poor skills) to 6 (Excellent skills)*.

a *N = 239 individual videos scored only once*.

b *N = 66 videos used for interrater reliability calculations*.

c *N = 140 videos scored with session goal 3 (not applicable to all sessions)*.

### Internal Consistency

The items within CAS CBT uses a 7-point ordinal Likert-scale, thus the reliability of the instrument was calculated using the Omega coefficient (McDonald, 1999). We examined the same domains as suggested by Bjaastad et al. (2016), computing the Omega's for the different key domains being evaluated during scoring. “CBT structure” (item 1–3) displayed an Omega of 0.85, whereas “Process and relational skills” (item 4–7) showed an Omega of 0.93. Since item 10 (session goal 3) could be rated *NA*, the number of assessed cases for the “Goals for the session” domain dropped to *n* = 140, consequently showing an omega of 0.70. When removing item 10, the coefficient improved to 0.76 (*n* = 238) (see Table 3). Omega if item deleted was also computed to assess any problem items within the scale, however, minimal differences were obtained in the overall Omega coefficient, which indicated that no specific problem items were found (see Table 3).

**Table 3:** Omega coefficients of the CAS-CBT.

**Key domains and items**	***N***	**ω**	**95% CI**	**ω if item deleted**	**Corrected item-total correlation**
**CBT structure** (Items 1–3)	239	0.85	(0.818–0.885)		
Item 1				0.85	0.42
Item 2				0.82	0.69
Item 3				0.84	0.78
**Process and relational skills** (Items 4–7)	239	0.93	(0.921–0.945)		
Item 4				0.84	0.54
Item 5				0.83	0.63
Item 6				0.84	0.53
Item 7				0.83	0.62
**Goals for the session** (Items 8–11)	140	0.70	(0.626–0.766)		
**Goals for the session** (Items 8, 9, and 11)	238	0.76	(0.722–0.914)		
Item 8				0.85	0.43
Item 9				0.85	0.50
Item 10				0.86	0.39
Item 11				0.82	0.78

*CI, Confidence Interval*.

### Correlations

Inter-item correlations were calculated for all 239 videos between the 11 items, ranging from *r* = 0.04, to *r* = 0.91. All correlations were significant, except for two, which was the correlation between item 6 and 10 (*r* = 0.17, *p* = 0.44), and item 8 and 10 (*r* = 0.04, *p* = 0.14), respectively (see Table 4).

**Table 4:** Polychoric correlations between items.

	**Item 1**.	**Item 2**.	**Item 3**.	**Item 4**.	**Item 5**.	**Item 6**.	**Item 7**.	**Item 8**.	**Item 9**.	**Item 10**.
**Item 2**.	0.58^**^									
**Item 3**.	0.71^**^	0.89^**^								
**Item 4**.	0.56^**^	0.57^**^	0.66^**^							
**Item 5**.	0.55^**^	0.54^**^	0.69^**^	0.72^**^						
**Item 6**.	0.51^**^	0.56^**^	0.69^**^	0.74^**^	0.82^**^					
**Item 7**.	0.56^**^	0.56^**^	0.75^**^	0.85^**^	0.88^**^	0.91^**^				
**Item 8**.	0.46^**^	0.58^**^	0.59^**^	0.43^**^	0.55^**^	0.51^**^	0.51^**^			
**Item 9**.	0.39^**^	0.60^**^	0.58^**^	0.40^**^	0.33^**^	0.34^**^	0.39^**^	0.24^**^		
**Item 10**.	0.24^**^	0.50^**^	0.44^**^	0.29^**^	0.24^**^	0.17	0.22^*^	0.04	0.42^**^	
**Item 11**.	0.57^**^	0.78^**^	0.83^**^	0.67^**^	0.67^**^	0.67^**^	0.73^**^	0.67^**^	0.71^**^	0.53^**^

*N = 239*.

* *Correlation is significant at the 0.05 level (2-tailed)*.

** *Correlation is significant at the 0.01 level (2-tailed). Items 1–3 indicate “Cognitive therapy structure,” items 4–7 indicate “Process and relational skills,” and items 8–11 indicate “Goals for the session.”*

The correlation between the mean total scores on Adherence and Competence showed a significant and strong association (*r* = 0.89, *p* < 0.01).

## Discussion

This study was conducted to examine the initial psychometric properties and of the CAS CBT (Bjaastad et al., 2016) and how it applies in a population of children receiving a preventive group intervention for symptoms of anxiety and/or depression. Previously the instrument has been used in clinical settings, mostly on therapy for child anxiety (Bjaastad et al., 2016; Villabø et al., 2018; Harstad et al., 2021; Jeppesen et al., 2021). Results from our study showed that this slightly modified version of the instrument had fair to good interrater reliability, acceptable reliability in terms of internal consistency, and expected inter-item correlations.

In general, the inter-rater reliability was good and within acceptable range, however, some of the inter-rater reliability scores were in the lower range <0.50, particularly for the items assessing process and relational skills (e.g., Positive reinforcement, Collaboration, Flexibility). This implies that either it was difficult to come to an agreement regarding these items, or there was something with the instrument that made it difficult to calibrate and reach consensus when scoring these items. As Lervik et al. (2021) also suggests, it is probably more difficult to score and interpret interpersonal relationships and the more abstract items, as opposed to more structural and concrete parts of a CBT-intervention (e.g., checking homework assignments, or putting up an agenda). Although the scoring team discussed the content and meaning of each item all along, a thorough operationalization beforehand could have provided even more accurate assessments.

More specifically, this domain regarding process and relational skills, consists of two adherence and two competence items assessing how the group leaders work to provide a positive and including environment. In general, the items cohered to such a large extent that it was difficult to estimate the scores from one another (scoring high on one item ultimately indicated a high score on the next item), especially within the different key domains. Further, the competence items were consistently evaluated based on a global assessment of two or three adherence items, where adherence seemed to explain much of the discrepancy within the different domains. During scoring, the raters would most often base the competence score on the adherence-ratings but emphasize them differently by allowing the topic of the particular session (e.g., problem solving) count more than a less pronounced theme (e.g., checking in on how the children are doing). This was a natural thing to do, since the main topic of a session required more time and effort from the group leaders. Evidently, this practice had an impact on the results and should be considered carefully upon further use of the instrument. In other studies, competence has shown to be rather difficult to agree upon (Barber et al., 2007; Hogue et al., 2008; Bjaastad et al., 2016). Providing a separate competence score for each item, could be one approach to avoid this issue. Alternatively, two separate measures for adherence and competence as proposed by other researches (e.g., Gutermann et al., 2015) could be conducted. However, both these suggestions would require a revision of the instrument and the scoring manual.

The high correlation between adherence and competence confirmed the overlap between these constructs. Similar results were found by Bjaastad et al. (2016), and in other measures as well (Shaw et al., 1999; Ginzburg et al., 2012). This lack of divergent validity between the constructs generally implies a strong relationship, however, some argue that raters have issues separating them from each other (Gutermann et al., 2015). According to the literature though, adherence and competence are conceptually different constructs, as adherence generally reflect the more quantifiable aspects of delivery (e.g., how often or to what extent the manual is followed), whereas competence includes more qualitative parts during delivery, such as relevant knowledge, skills, and attitudes (Kaslow, 2004). Investigating the association might be difficult though, because of the dependency between them (Perepletchikova et al., 2007).

Furthermore, high and significant correlations were also found between the items, reflecting a high dependency between the items as well. However, the adherence items rating the goals for the sessions showed particularly low inter-item correlations. The lack of correlation is not a total surprise given that the goals for the sessions are independent, indicating that you do not have to complete one goal before moving on to the next one. The different goals also vary from session to session in terms of content and extent, which was reflected by the uneven distribution of the response categories within these items. This could be related to issues, which we were unable to capture during scoring, such as the difference between missing (not completed at all) vs. a total lack of adherence to the program. One reason for this could be the transdiagnostic and comprehensive nature of the EMOTION manual, including many elements for each session. For the program developers, suggesting two or three main goals per session was challenging due to the extensiveness of the program content for each session. This could have affected the completion, and therefore also the scoring of these particular items regarding goals. This is also supported by the extant literature (Perepletchikova and Kazdin, 2005) where it is suggested that intervention characteristics may have an impact on program fidelity, as increased complexity is associated with lower scores on fidelity.

On the other hand, the low inter-item correlations may also highlight the uniqueness and program specificity being captured with the measure (Calsyn, 2000). This was also an argument to not conduct a CFA, which is generally used to measure whether an instrument assess the construct(s) it is intended to assess (Floyd and Widaman, 1995; Cohen and Swerdlik, 2009). The structure of the instrument is designed in a way that makes it possible to assess specific program activities, which are defined before using the instrument, with a tool that is applicable in different settings. The instrument also includes the item “Flexibility,” which focuses on how to adapt the program to the participants and the setting where it is employed. This could be recognized as “Flexibility within fidelity,” which has become highly relevant when delivering manual-based programs (Kendall et al., 2008). Providing an intervention adherently, but at the same time adapting the program to the service setting, and the participating children creates some issues in relation to assessment of fidelity and traditional instrument validation (Cohen and Swerdlik, 2009; Allen et al., 2018). This is because it might be debatable whether scores on items measuring adherence are the result of latent traits within therapists (like it is assumed in CFA or EFA), or whether scores are the result of group processes. If the latter is the case, factor analytic approaches may not be valid (Bollen and Lennox, 1991). This may also be the reason why studies fail to explain the relationship between fidelity and outcome (Webb et al., 2010; Fonagy and Luyten, 2019).

Flexibility within fidelity may, however, be particularly important within a group condition. Having up to 10 children in the group, could potentially contribute with some issues that do not arise during individual treatment and which we were not able to assess with the instrument in its current state (e.g., group dynamics, conflicts between the children, noise, etc.). This might have affected the completion of the session goals, and subsequently the overall scoring of the session. Future studies could adapt for this by including additional questions to assess group dynamics (e.g., group size, group setting) or other factors which might affect the completion of the sessions but are not directly linked to the group leaders' skills. Also, as this was a preventive intervention targeting children with symptoms of anxiety and depression, many of the children had unspecific symptoms and unestablished issues, which is more difficult to target compared to children in the clinical range with more specified problems. Hence, the session outcome could be more difficult to evaluate. This could also be the reason why the mean adherence score and the mean competence score, was somewhat lower in this study than the mean ratings of adherence and competence for similar interventions applied in outpatient clinics (Bjaastad et al., 2016; Villabø et al., 2018).

Although an overall acceptable to good reliability was obtained, we were not able to conduct analysis demonstrating the structural validity of the instrument due to the instrument design. Thus, there is a need to address these important dimensions of fidelity to better understand how they work and how interventions impact outcome (McLeod et al., 2009; Webb et al., 2010). Future research should therefore continue the development of fidelity measures with the goal of making them applicable to different service settings and interventions. Maybe even more important, future research should also focus on developing methods to validate these measures adequately. Thus, having a brief measure to assess if manual-based CBT interventions are delivered as intended in first line services, may help to create benchmark scores to establish and maintain program fidelity (McLeod et al., 2019). This could provide insightful knowledge regarding use of such programs, and potentially have implications for which programs should be offered to whom, and who should facilitate them. Focusing on fidelity is crucial to help determine the successfulness of a specific intervention in relation to outcomes (Durlak and DuPre, 2008). It may clarify if failures related to intervention outcomes reflect the intervention itself, or how it was implemented, which is critical in relation to implementation research in general and policy makers and decision makers especially.

## Limitations

The low ICCs are a limitation, suggesting inadequate agreement between raters. From a measurement perspective, though, it could also be due to the large number of response categories. In that way, the measure might benefit from a reduction of response categories and describing specific behavioral indicators for each of the items, which might help producing ratings that are more consistent between raters. Also, a large number of raters could have led to more disagreements regarding the items. Focusing on training and conducting accuracy testing frequently are necessary, as well as keeping the number of raters to a minimum.

Group leaders in EMOTION were rated as a unit, rather than as a primary and secondary group leader. This could have led to some disturbances during scoring and which group leader to focus on. Preferably, a unique score for the two individuals would be optimal to be able to detect any variation between the group leaders. Alternatively, assigning the group leaders' different roles as primary and secondary would also produce individual scores, which is not merged with the other group leader.

Also, due to practical reasons, we included only 20% of the sessions for video recording to minimize the workload on the group leaders as the intervention was being conducted on top of regular work. Another reason for reducing the number of sessions is related to security issues, as the group leaders had to bring the cameras with them each time they were recording. In the future though, recording all sessions and then randomly choosing 20% of the sessions should be considered as an alternative approach.

Further, it would have been beneficial to conduct other validation assessments. Perepletchikova and Kazdin (2005) have proposed some strategies to validate fidelity measures. These include testing the measure with two different treatments, giving the providers different training, or testing validity by correlating it to other measures (e.g., concurrent and discriminant validity). This was not feasible within the current study but should be considered in future studies.

## Conclusion

In conclusion, the CAS CBT (Bjaastad et al., 2016) is an attractive instrument to be used in settings outside clinical treatment, such as prevention of anxious and sad children. Although brief, the inclusion of both program specificity and more general overall scoring of CBT structure and principals shows a comprehensiveness of the instrument, capturing different elements within a CBT intervention. Some of the results though, such as low inter-rater reliability, indicated that the instrument should be improved. To increase applicability, the instrument should be further developed to fit even more within a group setting. Including questions assessing group size, dynamics, and other issues affecting the group might provide more accurate ratings. How to assess the session goal items adequately also needs further attention, both to capture whether low scores on the adherence is due to low group leader skills (not conducted), or that they were flexible in adapting the intervention to the needs of the participating children. Moreover, developing methods to assess fidelity measures should be further developed, as the traditional psychometric evaluation methods does not seem to fit adequately within the complex interaction between the providers of an intervention, context of delivery and recipients of the intervention.

## Data Availability Statement

The raw data supporting the conclusions of this article will be made available by the authors, without undue reservation.

## Ethics Statement

The studies involving human participants were reviewed and approved by The Regional Committee for Health and Medical Research Ethics (2013/1909/REK Sør-Øst). Written informed consent to participate in this study was provided by the participants' legal guardian/next of kin.

## Author Contributions

L-MPR took a central part in the data collection, assisted with the data analyses, and wrote the paper. JP collaborated with the design and writing of the study, and collaborated in writing and editing of the manuscript. BHH analysed the data and edited the manuscript, particularly the results and discussion parts. S-PN designed and wrote the study, and took part in the editing of the manuscript. KDM, FA, and AMS collaborated with the design and writing of the study, and editing of the manuscript. MM collaborated in the writing and editing of the manuscript. All authors have read and approved the manuscript.

## Conflict of Interest

KDM is one of the program developers, and therefore receives royalties from sales of the program manual. The remaining authors declare that the research was conducted in the absence of any commercial or financial relationships that could be construed as a potential conflict of interest.
